# Diagnostics for rare diseases

**DOI:** 10.1038/s43856-023-00247-3

**Published:** 2023-02-28

**Keywords:** Diagnosis, Paediatric research

## Abstract

Dr. Stephen Kingsmore is the President and CEO of Rady Children’s Institute for Genomic Medicine. His career as a physician-scientist has covered the implementation of genomic medicine approaches for rare genetic disorders. This has furthered the field of genomic medicine, in which genomic information about an individual is used as part of their clinical care to facilitate diagnosis or improve treatment. In this Q&A, we ask Dr. Kingsmore a series of questions about the challenges in diagnosing rare diseases and how diagnosis could be improved in the future.

**Figure:**
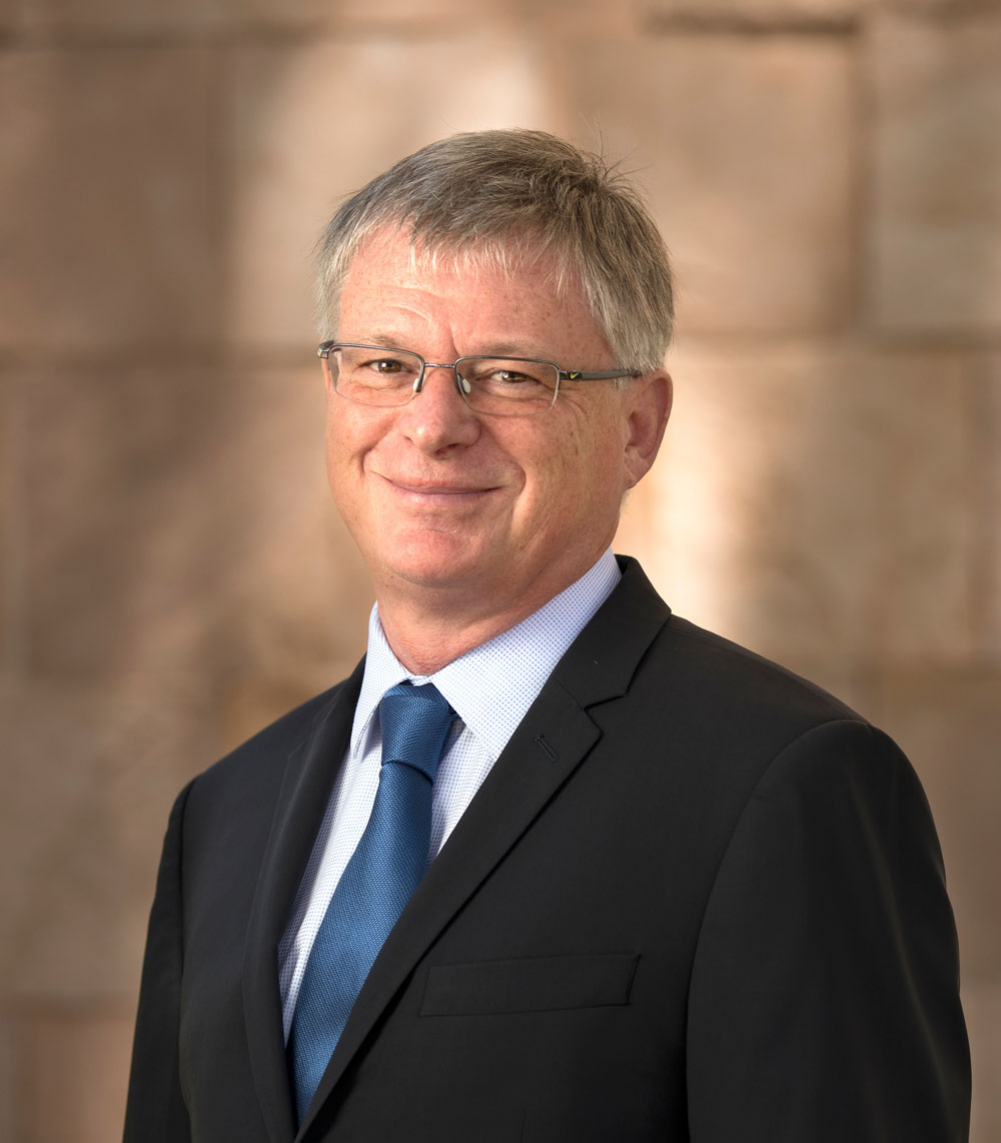
Photo by Earnie Grafton

Can you tell me a little bit about your background and your interest in this area?

I started my career as a physician-scientist attempting to identify rare Mendelian disease genes. My career changed when next-generation sequencing became possible in 2005. Since then, I’ve been working to invent and then implement genomic medicine for those same disorders. At first, in Santa Fe, New Mexico, at the National Center for Genome Resources, this involved pioneering work to decode individual human genomes and attempt to make sense of their medical relevance. Subsequently I moved to Children’s Mercy Hospital, Kansas City, to start a pediatric genomic medicine center. It was there that we developed rapid whole genome sequencing and found it to be very useful in newborns receiving intensive care both to provide empowering answers for parents and to identify effective therapeutic interventions. For the last 7 years I’ve been at Rady Children’s Hospital where we’re implementing genomic medicine on a much broader scale.

What are the challenges preventing diagnosis of rare diseases?

There are two main challenges today. The first is a lack of physician familiarity with individual rare genetic diseases. This comprises a lack of familiarity with their presenting symptoms and signs, with genome sequencing as a diagnostic tool, with treatments for these diseases, and with their outcomes. Collectively this has come to be known as the diagnostic odyssey, where parents of affected children see an estimated average of 7.3 specialist physicians over a period of 4.8 years before a diagnosis is made^1^. The sheer number of disorders is staggering. There are over 7300 of them in our compendium (Online Mendelian Inheritance in Man; https://www.omim.org/). Medical students and resident physicians are taught the mantra “if you hear hoof beats think of horses, not zebras”, coined in the 1940s by Dr. Woodward, a professor at the University of Maryland. What this means is when formulating a diagnosis of the probable cause of a child’s illness, always investigate the more common causes first. This works well for common diseases. It works horribly for rare diseases. The second is financial, which I’ll talk about below.

What are the best approaches currently for diagnosis of rare diseases?

Whole genome sequencing is, in the immortal words of JRR Tolkein, the “One ring to rule them all, one ring to find them, One ring to bring them all”. By decoding the entire genome it’s possible to examine it for variants responsible for almost all 7300 genetic diseases (and often to rule them out as causes of a child’s illness).

How does your work contribute to rare disease diagnostics?

Our institute, which is only 8 years old, was founded to “prevent, diagnose, treat, and cure childhood diseases through genomic and systems medicine research”. Our biggest project—called Tipping Point 10,000—was to catalyze the implementation of diagnostic whole genome sequencing for rare genetic diseases worldwide based on evidence in 10,000 published families. To accomplish this we have one of the clinical genome centers that is fastest to diagnose disease in the world. This year we’ll decode genomes from about 1200 families with affected children drawn from over 90 children’s hospitals throughout North America. The catalysis part involves engagement of many types of stakeholders to educate them about the benefits of early diagnosis and rapid diagnosis. Tragically, people still think of rare genetic diseases as something unmodifiable, when in fact there are effective therapies for well over 500 of them, and for all of them the power of diagnosis is immense for families. Physicians often think of genome sequencing as a test of last resort, when, in fact, the evidence is the opposite. Diagnostic genome sequencing when performed rapidly (with results in 3 days or less) saves over $10,000 per child receiving intensive care who is tested^2^.

Are there financial barriers to developing tests and how are these best overcome?

Rapid diagnostic whole genome sequencing is expensive. While there’s excitement about the “hundred dollar genome” it should be understood that that number is only the cost of the consumables needed when genomes are decoded at population scale. It’s 50–100 times that for a 3-day turnaround diagnostic-grade genome. There are several strategies to overcome the cost barrier. One is to publish cost effectiveness studies such as the one I mentioned above to demonstrate to stakeholders that diagnostic genome sequencing can lower healthcare costs for inpatients. This is slowly proving effective. There are now six US States with Medicaid policies to include rapid diagnostic whole genome sequencing and a national coverage policy by the large medical insurance companies Anthem/Blue Cross/Blue Shield and United Healthcare. In England and Wales there is a commitment to provide rapid diagnostic whole genome sequencing for all inpatient children who are suspected of having a genetic disease as part of the National Health Service. The other major strategy is to bring down the cost of testing. That’s also slowly happening.

How could diagnosis of rare diseases be improved in the future?

We recently published quite a provocative article in the journal JAMA Network Open^3^. By decoding the genomes of infants who had died we found that the leading cause of death was rare genetic diseases. Many of those diagnoses were made post-mortem from archived newborn blood spots. This was in San Diego where Rady Children’s Hospital is located and where diagnostic genome sequencing is probably more prevalent in sick infants than anywhere else. Even more shockingly, some of those deaths were avoidable as they were for the subset of about 500 genetic diseases for which effective therapies are available. This led to the epiphany that it would never be enough to decode the genomes of only those with symptoms that raised suspicion of having a genetic disease. What was needed was a highly cost effective, more scalable version of genome sequencing to screen all babies at birth for those 500 or so diseases. In August 2022, we published proof that this was possible^4^. We and about ten other groups worldwide are starting clinical trials of newborn screening by genomic sequencing. The goal is to identify and treat preventable genetic diseases at or before symptom onset. Furthermore, we hope to partner this with the companies who are developing new medicines for rare genetic diseases so that the time interval between new drug approval and finding the population of affected children is minimized.

Is diagnosis of rare diseases being adopted in other countries?

What started as a US experience with diagnostic rapid whole genome sequencing for hospitalized infants and children has now been recapitulated in at least a dozen countries. Physicians in Australia, for example, have published similar cost effectiveness data to us. Neonatal and pediatric intensive care units are found worldwide and almost universally have a very different cost:benefit perspective than other healthcare delivery situations. I am very excited about the possibility of the newborn screening version of genomic sequencing reaching a price point that makes economic sense for the 140 million babies born each year who receive at least one standard newborn screening test currently.
