# Author Correction: Diagnostics for rare diseases

**DOI:** 10.1038/s43856-023-00285-x

**Published:** 2023-04-13

**Keywords:** Diagnosis, Paediatric research

Correction to: *Communications Medicine* 10.1038/s43856-023-00247-3, published online 28 February 2023.

There was a mistake in the first sentence of this article. The job description for Dr Kingsmore read ‘Dr. Stephen Kingsmore is President/CEO of Rady Children’s Hospital’ but should have read ‘Dr. Stephen Kingsmore is the President and CEO of Rady Children’s Institute for Genomic Medicine’. The mistake has been corrected in the HTML and PDF versions of the article.

